# Supplementary material for: From sequence to enzyme mechanism using multi-label machine learning
Source: BMC Bioinformatics. 2014 May 19;15:150. doi: 10.1186/1471-2105-15-150 (PMC4229970; doi:10.1186/1471-2105-15-150)
Supplement: Additional file 2 — Java code of ml2db. Additional file ml2db_code.tar.gz contains the Java source code to run the multi-label machine learning experiments and save the results to database. The code’s Javadoc is included. [file 1471-2105-15-150-S2.zip › additional file 2/ml2db/ml2db/doc/mulan/evaluation/loss/RankingLossFunction.html]

RankingLossFunction


JavaScript is disabled on your browser.


- Overview
- Package
- Class
- Use
- Tree
- Deprecated
- Index
- Help

- Prev Class
- Next Class

- Frames
- No Frames

- All Classes

- Summary:
- Nested |
- Field |
- Constr |
- Method

- Detail:
- Field |
- Constr |
- Method


mulan.evaluation.loss

## Interface RankingLossFunction

- All Superinterfaces:
  :   MultiLabelLossFunction

  All Known Implementing Classes:
  :   ErrorSetSize, IsError, OneError, OneMinusAveragePrecision, RankingLoss, RankingLossFunctionBase

  ---

    

  ```
  public interface RankingLossFunction
  extends MultiLabelLossFunction
  ```

  Interfance for ranking loss functions

  Version:
  :   2010.11.10

  Author:
  :   Grigorios Tsoumakas

- - ### Method Summary

    Methods

    | Modifier and Type | Method and Description |
    | --- | --- |
    | `double` | `computeLoss(int[] ranking, boolean[] groundTruth)` Computes the ranking loss function |

    - ### Methods inherited from interface mulan.evaluation.loss.MultiLabelLossFunction

      `computeLoss, getName`

- - ### Method Detail


    - #### computeLoss

      ```
      double computeLoss(int[] ranking,
                       boolean[] groundTruth)
      ```

      Computes the ranking loss function

      Parameters:
      :   `ranking` - the ranking of the learner for an example
      :   `groundTruth` - the ground truth of the example

      Returns:
      :   the value of the loss function


- Overview
- Package
- Class
- Use
- Tree
- Deprecated
- Index
- Help

- Prev Class
- Next Class

- Frames
- No Frames

- All Classes

- Summary:
- Nested |
- Field |
- Constr |
- Method

- Detail:
- Field |
- Constr |
- Method
